# Impact of preformed donor-specific anti-HLA antibodies on pancreatic islet transplantation outcomes: do they matter as they do in solid organ transplantation?

**DOI:** 10.3389/fimmu.2026.1909238

**Published:** 2026-08-12

**Authors:** Ibrahim Tawhari, Fatmah Yamani, Manal Alotaibi, Mesfer Aldosari, Hany El Hennawy, Mohammed Tawhari

**Affiliations:** 1 Department of Internal Medicine, College of Medicine, King Khalid University, Abha, Saudi Arabia; 2 Department of Internal Medicine, Division of Nephrology, King Faisal Specialist Hospital & Research Center, Jeddah, Saudi Arabia; 3 Department of Internal Medicine, College of Medicine, Umm Al-Qura University, Mecca, Saudi Arabia; 4 Department of Surgery, Section of Transplantation, King Abdulaziz Medical City, Ministry of National Guard Health Affairs, Jeddah, Saudi Arabia; 5 King Abdullah International Medical Research Center, Jeddah, Saudi Arabia; 6 College of Medicine, King Saud Bin Abdulaziz University for Health Sciences, Riyadh, Saudi Arabia; 7 Department of Medicine, Division of Nephrology, King Abdulaziz Medical City, Ministry of National Guard Health Affairs, Riyadh, Saudi Arabia; 8 King Abdullah International Medical Research Center, Riyadh, Saudi Arabia

**Keywords:** allosensitization, antibody-mediated rejection, anti-HLA antibody, *de novo* DSA, donor-specific antibody, pancreatic islet transplantation, preformed DSA, type 1 diabetes

## Abstract

**Background:**

Preformed donor-specific anti-HLA antibodies (DSA) are a major risk factor for antibody-mediated rejection and graft failure in kidney, heart, lung, and other vascularized solid organ transplants. Their clinical significance in pancreatic islet transplantation, however, remains uncertain.

**Methods:**

We conducted a SANRA-guided narrative review using a focused PubMed search (January 1, 2000, to June 9, 2026) supplemented by citation chasing. Of 104 records identified, 11 directly evaluating islet transplantation formed the core evidence base, complemented by 38 references providing mechanistic, immunologic, and solid-organ context.

**Results:**

The available islet-specific evidence is dominated by small single-center studies, registry analyses, and case series, with few reports directly evaluating preformed DSA. Across these reports, preformed DSA have not shown the consistent adverse effect expected from kidney transplantation, but the evidence is insufficient to establish safety. In the largest focused cohort, Piemonti et al. reported no clear association between preformed DSA and graft failure, whereas post-transplant antibody evolution was more closely linked to impaired graft survival. Brooks et al. similarly found rapid graft dysfunction after *de novo* DSA, while Pouliquen et al. observed late *de novo* DSA without uniform graft loss. Registry and failure cohorts show that post-transplant allosensitization is clinically important, especially after immunosuppression withdrawal or repeat transplantation. A proposed mechanistic model is that portal infusion, low tissue mass, early instant blood-mediated inflammatory reaction, endothelial replacement, and non-vascularized graft architecture may reduce the immediate impact of pre-existing DSA while leaving the graft vulnerable to evolving humoral memory.

**Conclusions:**

The limited evidence is insufficient to support a universal policy of automatic exclusion of otherwise suitable islet candidates solely because of low-level preformed DSA; nor does it establish that such antibodies are safe. Because the evidence derives mainly from small single-center cohorts, registry analyses, and case series, this conclusion is provisional and may not be generalized across programs. *De novo* DSA represent a distinct post-transplant risk and may be a more clinically informative warning signal. Standardized MFI interpretation, complement-binding assays, longitudinal monitoring, and harmonized outcomes are needed.

## 1 Introduction

Type 1 diabetes (T1D) remains a global cause of early mortality, severe glycemic instability, and treatment burden. The International Diabetes Federation estimates that approximately 589 million adults aged 20–79 years are living with diabetes worldwide, and this number is projected to rise to 853 million by 2050 (1). For T1D specifically, modelling estimated approximately 8.4 million people living with T1D in 2021, with projected prevalence of 13.5-17.4 million by 2040 (2). For most patients, intensive insulin delivery, continuous glucose monitoring, and structured education reduce the risk of severe hypoglycemia and long-term complications. A clinically important minority nevertheless experience recurrent severe hypoglycemia, impaired awareness of hypoglycemia, and severe glycemic instability despite optimized medical therapy; these syndromes are associated with trauma, cardiac arrhythmia, fear of hypoglycemia, profound impairment of quality of life, and excess mortality (3).

Allogeneic pancreatic islet transplantation was developed to restore endogenous insulin secretion without whole-organ pancreas surgery. The Edmonton protocol demonstrated reproducible insulin independence using steroid-free immunosuppression and adequate islet mass, changing the therapeutic landscape for adults with T1D complicated by recurrent severe hypoglycemia or glycemic lability (4). Subsequent follow-up showed that although insulin independence often diminishes over time, partial graft function may persist, as evidenced by detectable C-peptide secretion, improved glycemic control, and protection against severe hypoglycemia after return to exogenous insulin (5). Registry analyses show progressive improvement in outcomes: the Collaborative Islet Transplant Registry reported increasing three-year insulin independence across eras, from 27% in 1999–2002 to 44% in 2007-2010, together with durable C-peptide secretion and hypoglycemia protection (6). More recent CITR analyses and European cohorts confirm that primary graft function predicts five-year outcomes and that modern programs can achieve clinically meaningful long-term metabolic benefit (7, 8). Randomized and comparative studies, including TRIMECO, reinforced the value of islet transplantation for severe hypoglycemia and glycemic instability in highly selected patients (9–12). Regulatory recognition has also changed: the US Food and Drug Administration approved the first allogeneic pancreatic islet cellular therapy for adults with T1D and recurrent severe hypoglycemia in 2023 (13).

Yet islet transplantation remains immunologically distinct. Unlike kidney, heart, and lung transplantation, the islet cell graft is not a vascularized organ with donor endothelium lining a continuous microcirculation. Islets lodge in the hepatic sinusoids and undergo substantial early innate attrition before adaptive alloimmunity matures (14–17). Therefore, the standard solid-organ dogma that preformed DSA mark a recipient as high risk and may directly trigger ABMR cannot be assumed to apply without qualification.

Terminology is used hierarchically in this review. Anti-HLA antibody is the broadest term. Sensitization denotes detectable anti-HLA reactivity and is commonly summarized by PRA or cPRA, which estimate the breadth of reactivity but do not establish donor specificity. DSA are antibodies directed against HLA antigens present in a specific donor; preformed DSA are detectable before infusion, whereas *de novo* DSA arise after transplantation.

The central paradox is that preformed DSA, which are strongly associated with adverse outcomes in kidney transplantation, have yielded inconsistent associations in islet transplantation, whereas *de novo* DSA appear to be more consistently associated with graft attrition, immunosuppression failure, or alloimmune memory (18–22). This review critically synthesizes the evidence for preformed and *de novo* DSA in pancreatic islet transplantation, compares these patterns with solid-organ transplantation, and proposes a conceptual framework for clinical practice and future cellular replacement therapies.

## 2 Methods: narrative review with systematic search methodology

This narrative review was structured according to SANRA criteria: the purpose was explicitly defined, a focused literature search was performed, referencing was prioritized, scientific reasoning and balance were emphasized, and uncertainty was made explicit (23).

The search period was January 1, 2000, through June 9, 2026. PubMed was searched using combinations of: pancreatic islet transplantation, islet-after-kidney transplantation, donor-specific antibody, anti-HLA antibody, preformed DSA, pre-existing DSA, *de novo* DSA, allosensitization, antibody-mediated rejection, HLA sensitization, C-peptide, insulin independence, HbA1c, severe hypoglycemia, graft survival, and graft failure. Searches were supplemented by backward and forward citation chasing of CITR, Edmonton protocol, GRAGIL/CIT Consortium, transplant immunology, and solid-organ DSA publications. Embase, Scopus, Web of Science, and CENTRAL were not searched directly for this article, so the evidence synthesis should be read as a focused PubMed-based narrative review rather than a comprehensive systematic review.

Eligible records included human islet transplantation studies reporting HLA antibodies, DSA, PRA, crossmatching, sensitization, graft outcomes, graft failure, or humoral rejection; mechanistic studies that clarified islet alloimmunity; and selected solid-organ studies needed to contextualize the biologic contrast. Exclusion criteria were non-human studies without direct mechanistic relevance, purely technical assay papers without transplantation outcomes, commentary without original data unless needed for guidance, and solid-organ DSA reports not used for comparison. Outcomes of interest were insulin independence, C-peptide persistence, HbA1c, severe hypoglycemia, graft survival, graft loss, repeat transplantation, allosensitization, rejection, and long-term metabolic function.

Search reporting: a focused PubMed search combining islet transplantation with anti-HLA antibody, donor-specific antibody, and sensitization terms (January 1, 2000-June 9, 2026) identified 104 records. At title and abstract screening, 76 records were excluded: 42 non-human or experimental-model studies, 15 reviews or editorials without primary data, and 19 records that did not report anti-HLA antibody, DSA, panel-reactive antibody, or sensitization outcomes in islet recipients. The remaining 28 reports were assessed in full text, of which 17 were excluded because they described autologous islet transplantation without alloimmune relevance, reported overlapping or duplicate cohorts, or provided DSA or sensitization data only in a solid-organ or mechanistic context. Eleven direct islet-transplant reports met all eligibility criteria and constituted the core evidence base (18–22, 24–28, 41) (**Table 1**). A further 38 mechanistic, solid-organ, epidemiologic, and background references were identified through backward and forward citation chasing and targeted searches and were cited to provide biological and clinical context, yielding 49 references in total (**Supplementary Figure 1**).

**Table 1:** Characteristics of included islet-transplant studies addressing anti-HLA antibodies, DSA, or sensitization.

*Author*	Year	Country	Design	Patients	Follow-up	Preformed DSA prevalence	Assay	Endpoints	Main findings	Evidence appraisal
*Lobo*	2005	USA	Case report	1	Early post-transplant	Not DSA-focused; anti-HLA class I after allogeneic islets	Anti-HLA antibody testing	Allosensitization	Demonstrated development of anti-HLA antibodies after allogeneic islet transplantation	Low; early assay era (24)
*Cardani*	2007	USA	Clinical cohort	66	Variable	Allosensitization focus	Panel/HLA antibody assays	Allosensitization after islet transplant	Showed that islet transplantation can sensitize recipients and affect future donor options	Low to moderate; descriptive (26)
*Ferrari-Lacraz*	2008	Switzerland	Combined kidney-islet series	37 (islet-alone 8, islet-after-kidney 13, simultaneous islet-kidney 16)	Short clinical follow-up	Low sensitization signal	Anti-HLA antibody testing	Anti-HLA sensitization	Reported low risk of anti-HLA sensitization after combined kidney and islet transplantation	Low; small cohort (27)
*van Kampen*	2005	Netherlands	Immunologic cohort	3 (each received 3 sequential islet grafts)	Post-infusion monitoring	Repeated mismatches assessed	Alloreactivity/HLA antibody assessment	Alloreactivity to repeated mismatches	Highlighted relevance of repeated HLA mismatches for subsequent infusions	Low; early cohort (25)
*Kessler*	2009	France	Case report	1	Clinical course after single infusion	Preformed class II DSA that increased post-transplant	HLA antibody testing	Graft dysfunction and rescue therapy	HLA sensitization coincided with graft dysfunction; rituximab/IVIG associated with recovery	Very low; proof of concept (41)
*Naziruddin/CITR*	2012	International registry	Registry analysis	303 islet-alone recipients from 1999-2008	Registry follow-up	Pretransplant PRA not predictive; post-transplant PRA >20% important	PRA/HLA class I sensitization	Graft failure	Post-transplant PRA >20% associated with higher graft-failure risk; pretransplant PRA not independently predictive	Moderate; registry but class I focus (28)
*Piemonti*	2013	Italy	Single-center cohort	59	Longitudinal	29 preformed DSA; 24 *de novo* DSA	Alloantibody and autoantibody monitoring	Graft failure, metabolic outcomes	Primary adverse signal was a post-transplant increase in DSA and/or beta-cell autoantibodies; pretransplant PRA >15% was an independent risk factor for antibody increase; preformed DSA alone was not the dominant signal	Moderate; key cohort, limited by size, assay-era variation, and heterogeneous donor exposure (18)
*Brooks*	2015	UK	Prospective/observational cohort	16 participants; 26 islet grafts	12 months and serial monitoring	*De novo* DSA after 5 grafts (19%); preformed focus limited	HLA antibody monitoring, MMTT	Graft function	*De novo* DSA associated with rapid loss of graft function	Moderate; small but temporal signal (19)
*Pouliquen*	2017	France	Clinical cohort	42	Longitudinal; median dnDSA appearance 25.5 months	1 preformed DSA; 12 *de novo* DSA	Anti-donor HLA antibody monitoring	Graft function/graft loss	Late *de novo* DSA not uniformly associated with graft loss, even high MFI	Moderate; nuanced signal (20)
*Rios*	2021	USA	Long-term failure cohort	35 ITx recipients; 17 with graft failure and 18 with persistent graft function	Long-term post-failure follow-up; allosensitization persisted for 7–15 years in some patients	Post-failure sensitization/DSA in graft-failure subgroup	PRA, cPRA, DSA	Persistence of allosensitization	Among 35 recipients, 17 developed graft failure; durable allosensitization was especially evident after immunosuppression withdrawal	Moderate; important long-term data with subgroup denominator clarified (21)
*Maanaoui*	2022	France	Illustrative case-series	32 recipients analyzed with available sera; 7 had DSA at any time	Clinical follow-up	7/32 DSA-positive; 2 preformed only, 1 both preformed and *de novo*, 4 *de novo* only	SAB MFI, donor specificity	Insulin independence/graft survival	Selected preformed-DSA recipients had favorable outcomes; only 3/32 had preformed DSA, so wording should not imply that preformed DSA were common	Low to moderate; selected illustrative case-series with explicit denominator (22)

Evidence appraisal in **Table 1** is a narrative study-level judgment based on design, sample size, assay era, donor-specific assignment, longitudinal follow-up, and outcome ascertainment; it is not a validated risk-of-bias score.

### 2.1 Biology of islet alloimmunity

**Figure 1:**
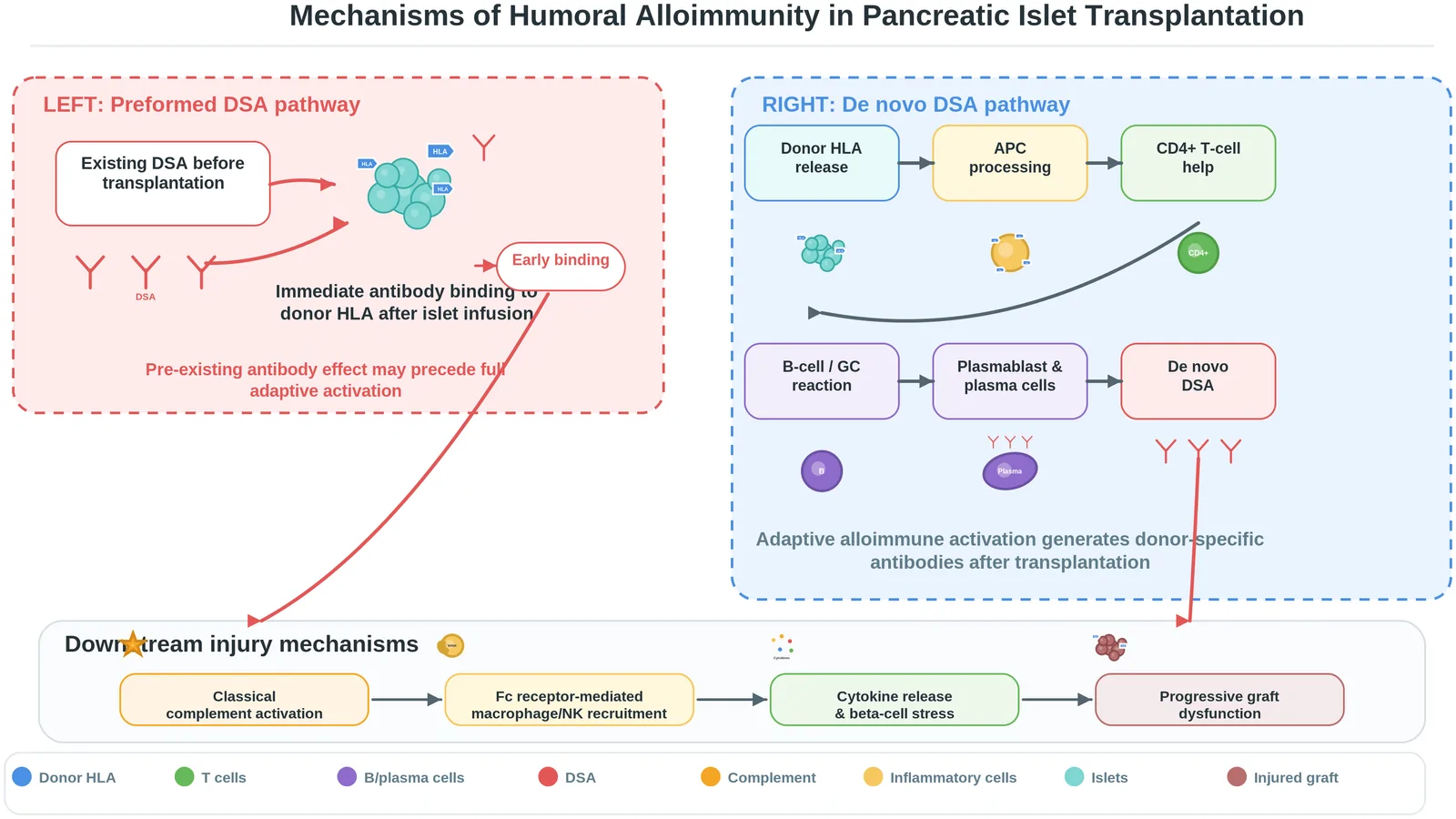
Original schematic of humoral alloimmunity in pancreatic islet transplantation, distinguishing preformed from *de novo* DSA and showing potential convergent injury pathways. Direct and indirect allorecognition and DSA formation are supported by published evidence; the integrated sequence and relative contribution of pathways are the authors’ conceptual model.

The adaptive response involves both direct and indirect allorecognition. Donor antigen-presenting cells and donor HLA molecules stimulate recipient T cells, while recipient antigen-presenting cells present donor peptides to T cells which activate B cells. Memory T cells, B cells, plasma cells, and alloantibody responses may then target donor HLA antigens. Because islet recipients frequently require infusions from multiple donors to achieve adequate islet mass, each sequential transplant increases cumulative HLA sensitization. Repeat infusions may restore metabolic function but can also prime a more vigorous alloimmune response against subsequent donor antigens (24–28). Autoimmunity constitutes an additional and important complicating factor: cellular and humoral autoimmunity against beta-cell antigens may coexist with alloimmunity, confounding attribution of graft dysfunction to DSA alone (18, 29, 30). Mechanistic work on non-classical allorecognition and contemporary reviews of islet rejection underscore that DSA formation and rejection diagnosis in cellular grafts may not follow the same pathways as vascularized-organ ABMR (31, 32) (**Figure 1**).

**Figure 2:**
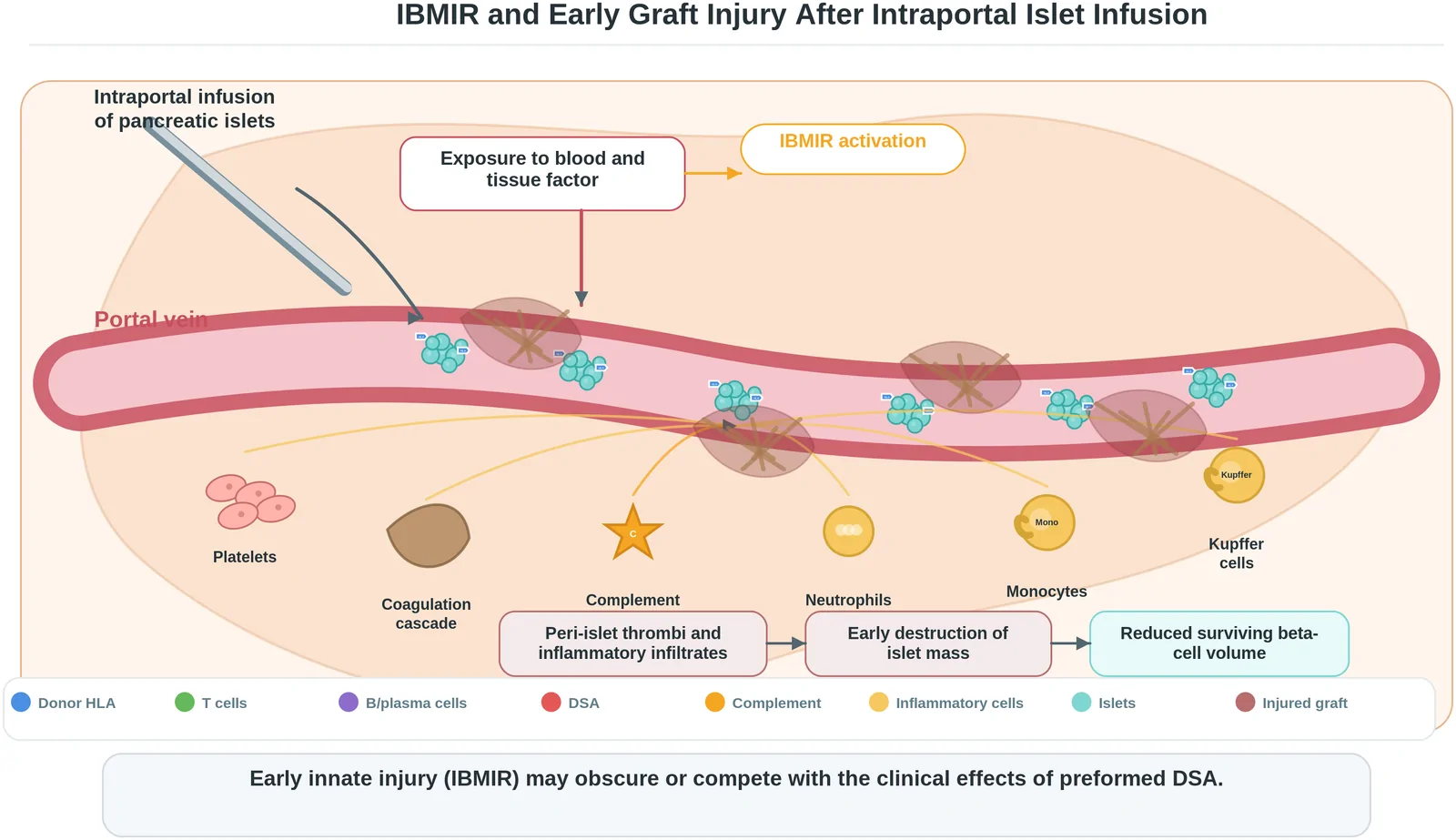
Original schematic of IBMIR and early graft injury after intraportal islet infusion, emphasizing blood exposure, innate effector activation, peri-islet thrombi, inflammation, and early beta-cell mass loss. IBMIR components are evidence-supported; their proposed interaction with the clinical effect of preformed DSA is conceptual.

Islet allograft injury begins before a classical adaptive rejection response can be organized. On contact with portal blood, isolated human islets trigger the instant blood-mediated inflammatory reaction (IBMIR), a thromboinflammatory process characterized by platelet activation and consumption, tissue factor-driven coagulation, complement activation, leukocyte recruitment, and fibrin deposition around islets (14–17). This early thromboinflammatory response is not confined to experimental observations; it helps explain why only a fraction of infused islet mass engrafts and why early graft function depends on islet quality, exposure to portal flow, anticoagulation, innate immunity, and local hepatic inflammation (**Figure 2**).

Antibody-mediated islet injury may differ from vascularized-organ ABMR. In a kidney allograft, DSA binds donor endothelial HLA in a continuous microvascular bed, fixes complement, recruits innate immune effectors, and produces microvascular inflammation. In islet transplantation, early donor endothelium may be lost or replaced, and islets become embedded within the recipient hepatic parenchyma via portal tributaries rather than maintaining an intact donor vascular tree (33). This anatomic distinction is a plausible reason why preformed DSA may not behave with the same deterministic toxicity as in kidney transplantation. However, this does not make humoral immunity irrelevant; it may suggest that the timing, antigen load, complement-fixing capacity, class specificity, and persistence of DSA matter more than a single pretransplant positive assay.

### 2.2 HLA matching and antibody testing in islet transplantation

HLA matching has historically been less stringent in islet transplantation than in kidney transplantation. Several factors contributed to this practice: life-saving urgency is usually lower than in deceased-donor kidney allocation, islets are often pooled or administered from multiple donors, donor availability is limited, immediate islet quality and islet mass strongly influence success, and early IBMIR and engraftment failure compete with adaptive alloimmunity as causes of graft loss (6, 15–18).

Contemporary practice increasingly integrates donor-specific antibody assignment, unacceptable antigen listing, class I and class II antibody characterization, calculated PRA, complement-binding assays in selected cases, and virtual crossmatch. SAB assays are highly sensitive but semi-quantitative; MFI is affected by antigen density, bead lot, serum treatment, prozone, complement interference, and laboratory thresholds (35). Inter-laboratory comparability is further limited by differences in platforms, serum pretreatment, positivity cutoffs, and reporting conventions. Accordingly, an MFI value does not represent a standardized biologic concentration and should not serve as a universal exclusion criterion. This caveat is especially important in islet transplantation, where the same numeric MFI may not correspond to the same graft injury risk observed in kidney transplantation.

Class II antibodies, particularly HLA-DQ DSA, deserve special attention. In kidney and lung transplantation, *de novo* DQ antibodies are disproportionately associated with chronic injury (34, 35, 38). In islet transplantation, *de novo* class II responses may reflect late alloimmune maturation, immunosuppression reduction, or antigenic recall after repeated donor exposure (19–22). Crossmatch-positive or highly sensitized candidates should therefore be evaluated individually, considering antibody specificity, strength, complement binding, historic sera, donor options, kidney-transplant status, need for repeat islet infusion, and the consequences of further sensitization.

### 2.3 Preformed donor-specific anti-HLA antibodies

Preformed DSA are clinically intuitive risk markers because they represent recipient humoral memory against donor HLA before infusion. In vascularized solid organs, especially kidney transplantation, they predict early ABMR and inferior graft survival (35–37, 39). The islet literature is more equivocal. Reported preformed DSA prevalence has ranged widely, from absent in some cohorts to approximately half of recipients in selected series, reflecting differences in candidate selection, assay era, MFI thresholds, donor assignment, and whether historic or current sera were analyzed (18, 22) (**Table 2**).

**Table 2:** Clinical impact of preformed DSA in pancreatic islet transplantation.

Study	n	DSA frequency	MFI	Insulin independence	Graft survival	C-peptide	Significance	Conclusions
*Naziruddin/CITR (* 28 *)*	303 islet-alone recipients	Pretransplant PRA, not DSA-specific	PRA rather than SAB MFI	Not primary endpoint	Post-transplant PRA >20% associated with graft failure; pretransplant PRA not predictive	Not primary	Pretransplant sensitization signal weaker than post-transplant sensitization	Pretransplant humoral memory alone did not define outcome in registry data
*Ferrari-Lacraz (* 27 *)*	37 (8 islet-alone, 13 islet-after-kidney, 16 simultaneous islet-kidney)	Low reported sensitization risk	Not standardized to modern MFI	Not central	No strong adverse signal reported	Not central	Descriptive	Combined kidney-islet recipients on maintenance immunosuppression may differ from islet-alone recipients
*Piemonti (* 18 *)*	59	29 with preformed DSA; 24 with *de novo* DSA	MFI thresholds not standardized (assay-era dependent)	Preformed DSA not clearly associated with insulin-independence failure	Post-transplant antibody increase (DSA and/or autoantibodies) was the primary adverse signal; isolated preformed DSA was not clearly associated with graft failure	Autoantibody and alloantibody monitoring included	Pretransplant PRA >15% independently predicted post-transplant antibody increase; *de novo*/post-transplant antibody evolution was more clinically informative than preformed DSA alone	Largest focused cohort supports individualized evaluation rather than automatic exclusion, while recognizing antibody evolution as a risk signal
*Pouliquen (* 20 *)*	42	1 preformed DSA	MFI reported for dnDSA; preformed rare	Insufficient preformed events	No conclusion possible for preformed DSA	Not decisive	Underpowered for preformed DSA	Useful mainly for *de novo* DSA timing/outcomes
*Maanaoui (* 22 *)*	32 recipients analyzed	7/32 had DSA at any time; 3/32 had preformed DSA (2 preformed only, 1 both preformed and *de novo*)	Some MFI >10,000	Favorable outcomes reported in selected preformed-DSA recipients	Preformed DSA cases could maintain function; late *de novo* DSA was the unfavorable pattern	C-peptide/metabolic function favorable in cases	No population inference; denominator must be stated	Preformed DSA can be compatible with success in selected cases, but were uncommon in the analyzed cohort

Piemonti et al. conducted the most extensively cited focused analysis. In 59 islet recipients, 29 had preformed DSA and 24 developed *de novo* DSA during follow-up (18). The primary adverse signal should be framed as a combined post-transplant increase in DSA and/or beta-cell autoantibodies rather than as isolated preformed DSA alone. In that cohort, pretransplant PRA greater than 15% in either class I or class II was an independent risk factor for post-transplant antibody increase, while *de novo* antibody responses were more closely linked to impaired graft survival (18). This pattern is not proof of safety for preformed DSA; the cohort was limited, immunosuppression and donor exposure were heterogeneous, and residual confounding is likely. It nonetheless challenges the direct extrapolation of kidney-allograft paradigms to islet transplantation.

Maanaoui et al. extended this challenge through a single-center case-series analysis of 32 recipients with available sera across follow-up (22). Seven of 32 recipients had DSA at any time: 2 had preformed DSA only, 1 had both preformed and *de novo* DSA, and 4 had *de novo* DSA only; therefore, only 3 recipients had preformed DSA (22). Selected recipients with preformed DSA, including high-MFI antibodies, had favorable metabolic outcomes, including one patient with DSA against multiple donors and several MFI values above 10,000 (22). The denominator and selection should be explicit: this report supports the possibility of success in carefully selected preformed-DSA-positive recipients, but it does not show that preformed DSA were common or uniformly safe.

Earlier sensitization studies further contextualize this evidence base. Lobo and Cardani reported that anti-HLA antibodies and allosensitization can develop after islet transplantation (24, 26), while the CITR HLA class I analysis found that pretransplant PRA was not independently predictive of graft failure, whereas post-transplant PRA greater than 20% was associated with substantially higher graft-failure risk (28). Ferrari-Lacraz et al. demonstrated a low risk of anti-HLA sensitization in combined kidney and islet transplantation, suggesting that continuous immunosuppression and the immunologic context of the islet-after-kidney setting may attenuate humoral risk (27). van Kampen et al. raised a practical allocation question: repeated exposure to shared HLA mismatches during subsequent infusions may influence alloreactive memory and donor selection (25).

### 2.4 *De novo* DSA

**Figure 3:**
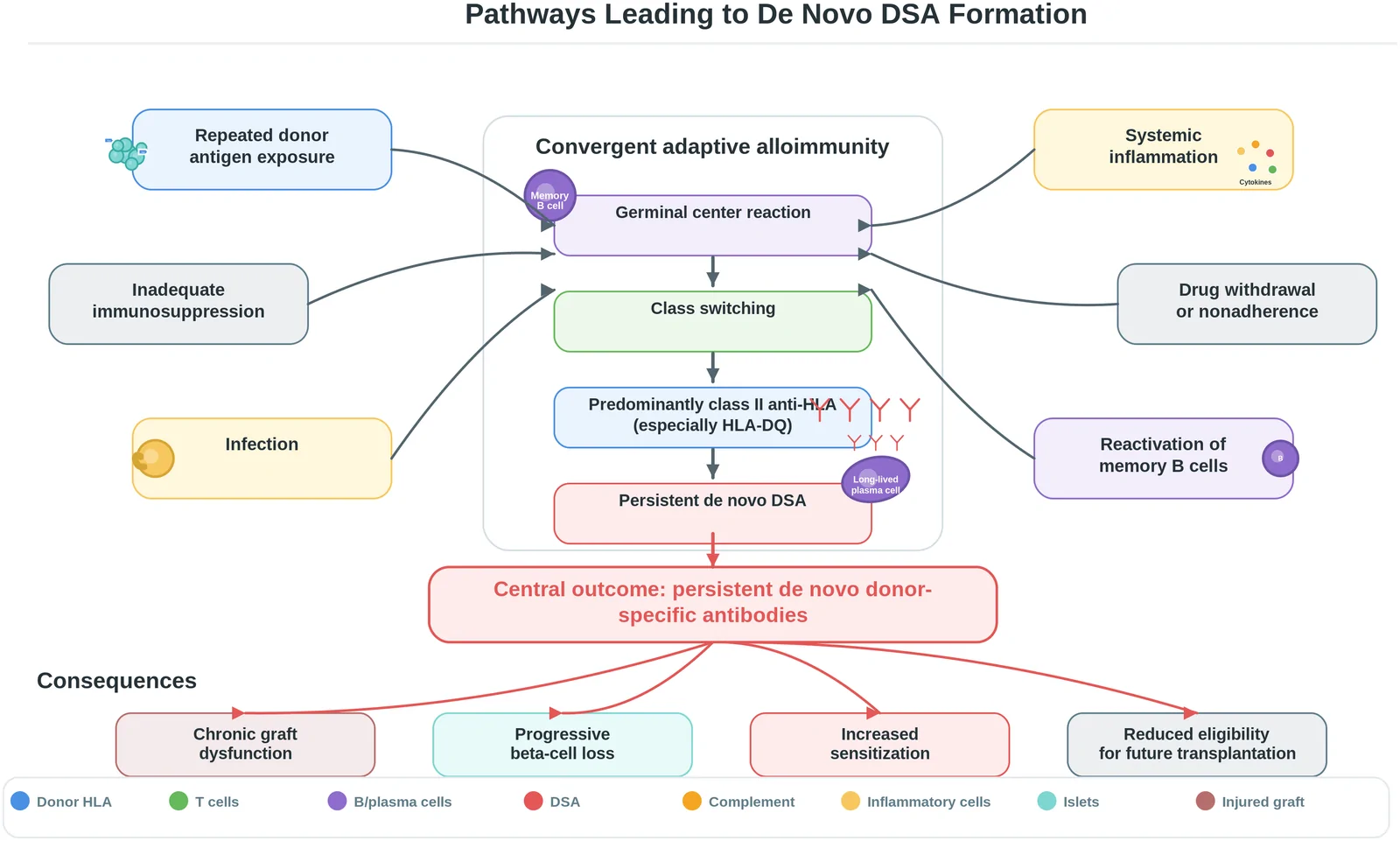
Original schematic of pathways leading to *de novo* DSA formation, showing potential triggers, germinal-center activity, class switching, persistent DSA, and clinical consequences. Individual elements are supported by transplant immunology literature; the sequence and weighting of pathways are conceptual.

*De novo* DSA represent an active alloimmune response after antigen exposure. They may arise after insufficient immunosuppression, drug minimization or withdrawal, repeat transplantation, infection-associated inflammation, memory B-cell activation, or ongoing antigen presentation from injured graft tissue. In islet transplantation, *de novo* DSA may be more informative than preformed DSA because they reflect the recipient-graft interaction after engraftment and because their emergence may coincide with cellular rejection, beta-cell stress, or progressive loss of functional islet mass (18–22) (**Figure 3**).

Brooks et al. reported 16 participants receiving 26 islet transplants under tacrolimus and mycophenolate-based immunosuppression with serial mixed-meal tolerance testing (MMTT) and HLA antibody monitoring. *De novo* DSA developed after five grafts, approximately 19% of grafts, and were associated with rapid loss of graft function by 12 months (19). Although small, this report is clinically informative because antibody emergence and functional decline were temporally concordant.

Pouliquen et al. studied 42 recipients and identified one recipient with preformed DSA and 12 with *de novo* DSA. *De novo* DSA appeared late, at a median of 25.5 months, and were not uniformly associated with graft loss, even when MFI exceeded 6000 (20). Rather than refuting Brooks et al., this study suggests heterogeneity: some *de novo* antibodies may be late biomarkers of immune exposure without immediate destructive effect, whereas others may be pathogenic, particularly if complement-binding, persistent, class II/DQ directed, or accompanied by worsening metabolic indices.

Rios et al. demonstrated why *de novo* allosensitization carries clinical significance even after graft failure. They evaluated 35 patients with T1D who received islet transplantation, including 17 with graft failure (13 islet-transplant-alone recipients and 4 islet transplant plus bone marrow-derived hematopoietic stem cell recipients) and 18 with persistent graft function (21). In those with graft failure, sensitization persisted for many years; many remained PRA-positive and several had DSA to prior donors, with cPRA values ranging from moderate to extremely high (21). This observation reframes the clinical question from short-term insulin independence to the lifetime transplantation trajectory: *de novo* DSA may jeopardize repeat islet infusion, kidney transplantation, pancreas transplantation, or future cellular products.

Kessler et al. provide proof of principle that humoral rejection can be clinically meaningful in islet transplantation. A recipient who achieved insulin independence after a single intraportal infusion developed graft dysfunction associated with HLA sensitization; rescue therapy with rituximab and intravenous immunoglobulin was followed by functional recovery (41). A case report cannot define incidence, but it supports the biologic plausibility of treatable humoral injury in selected patients (**Table 3**).

**Table 3:** Studies evaluating *de novo* DSA or post-transplant allosensitization.

Author	Year	Patients	Incidence	Target HLA	Timing	Outcomes	Conclusions
*Lobo (* 24 *)*	2005	1	Anti-HLA class I antibodies after transplant	Class I	Early post-transplant	Allosensitization	Allogeneic islet transplantation can provoke HLA antibodies
*Cardani (* 26 *)*	2007	66	Allosensitization described	Class I/II depending assay	Post-transplant	Future donor risk	Allosensitization is a meaningful downstream outcome
*Naziruddin/CITR (* 28 *)*	2012	303 islet-alone recipients	Post-transplant PRA >20% subset	Class I focus	After transplant	3.6-fold higher graft-failure risk reported for post-transplant PRA >20%	Post-transplant sensitization more prognostic than pretransplant PRA
*Kessler (* 41 *)*	2009	1	Preformed class II DSA that rose post-transplant, with graft dysfunction	Class II	After insulin independence	Graft dysfunction rescued by rituximab/IVIG	Proof that humoral injury may be clinically reversible
*Piemonti (* 18 *)*	2013	59	24 developed *de novo* DSA; 39/59 had post-transplant DSA and/or autoantibody increase	Not limited to one class	During follow-up	Combined post-transplant antibody increase predicted impaired outcomes; pretransplant PRA >15% was an independent risk factor for antibody increase	Key evidence that post-transplant antibody evolution, not preformed DSA alone, is the central adverse signal
*Brooks (* 19 *)*	2015	16 recipients; 26 grafts	5 grafts (19%) with dnDSA	Donor-specific HLA antibodies	Serial early monitoring	Rapid loss of graft function by 12 months	Strong temporal association despite small cohort
*Pouliquen (* 20 *)*	2017	42	12 recipients with dnDSA	Anti-donor HLA; no C3d complement-binding DSA	Median 25.5 months	No uniform graft loss, even MFI >6000	Shows heterogeneity and late noncatastrophic dnDSA
*Rios (* 21 *)*	2021	35 ITx recipients; 17 with graft failure and 18 with persistent graft function	Persistent post-failure DSA/PRA common in the graft-failure subgroup	Donor-directed antibodies to prior donors	After graft failure and immunosuppression withdrawal	Long-term sensitization, cPRA expansion	Post-failure DSA/PRA has major consequences for future transplantability; denominator clarified
*Maanaoui (* 22 *)*	2022	32 recipients analyzed; 7 DSA-positive at any time	4 *de novo* only and 1 both preformed and *de novo*; late *de novo* DSA emphasized as threat	Often class II in conceptual discussion	Late post-transplant	Graft loss in late dnDSA contexts	Supports long-term monitoring and careful distinction between preformed and *de novo* DSA

### 2.5 Preformed versus *de novo* DSA: a comparative synthesis

Preformed and *de novo* DSA are related but distinct clinical questions. Preformed DSA inform candidate and donor selection before infusion, whereas *de novo* DSA inform post-transplant surveillance, graft evolution, and future sensitization. The available evidence for preformed DSA is sparse and does not establish either safety or a uniform adverse effect; the *de novo* DSA literature is somewhat larger but remains observational and heterogeneous (18–22).

Several mechanisms could explain the asymmetry. First, preformed DSA encounter islets at the same time as IBMIR, hypoxia, complement activation, and coagulation; early graft loss may be dominated by innate thromboinflammation rather than by classical ABMR (14–17). Second, transplanted islets do not maintain a stable donor endothelial vascular bed comparable to a kidney microcirculation; endothelial chimerism and vascular remodeling may reduce the target for persistent anti-donor endothelial binding (33). Third, islet mass is low and dispersed, so a preformed antibody signal may be obscured by non-antibody determinants of engraftment, including islet dose, quality, portal pressure, and peri-infusional inflammation. Fourth, *de novo* DSA may mark sustained antigen recognition by a competent adaptive immune system after the graft has survived long enough to become immunologically detectable. These mechanisms are biologically plausible but remain inferential and should not be read as demonstrated protection from preformed DSA.

**Figure 4:**
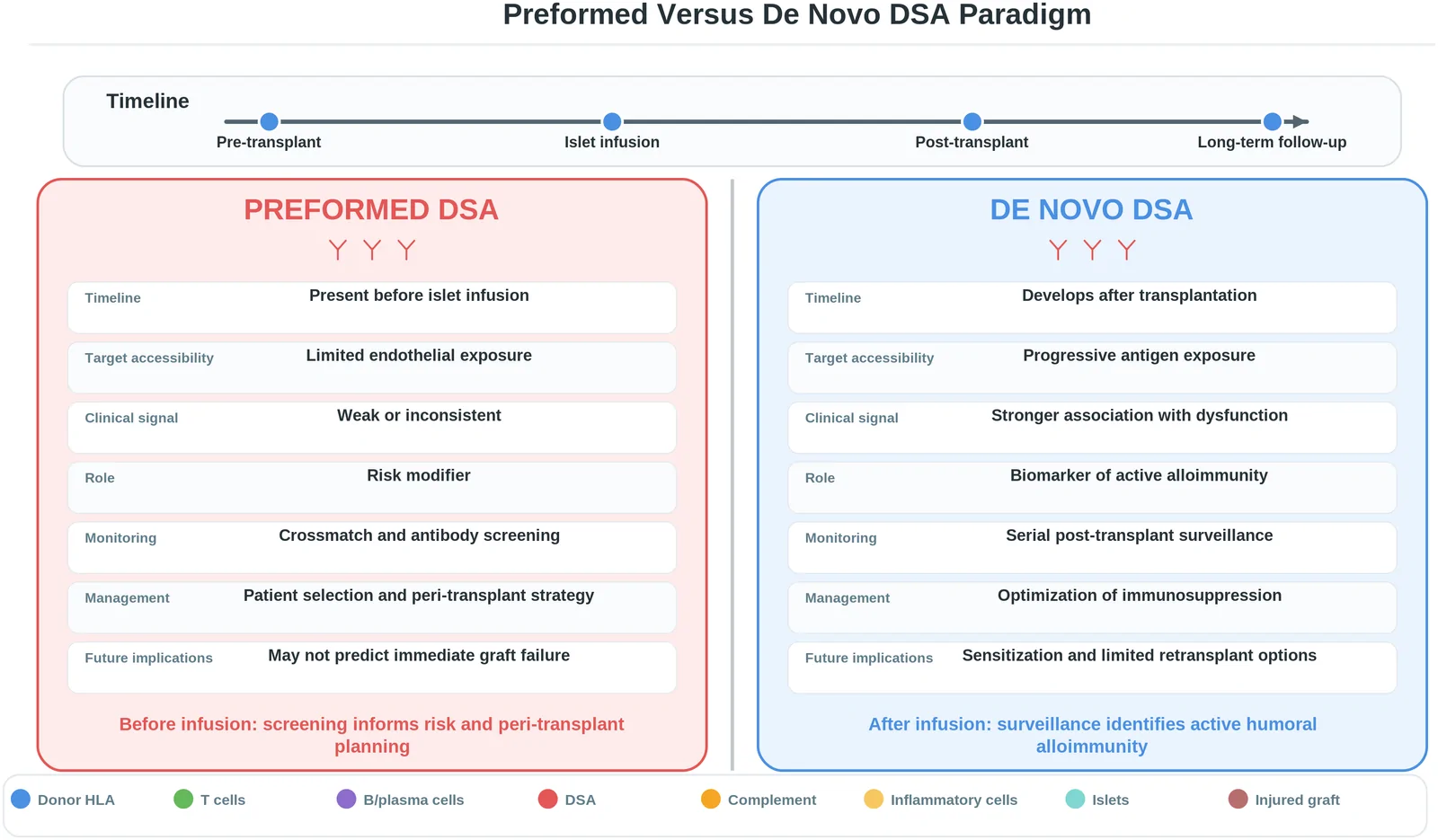
Original conceptual synthesis comparing preformed and *de novo* DSA in pancreatic islet transplantation across timing, target accessibility, monitoring, management, and future implications. The comparison is derived from the reviewed evidence but has not been prospectively validated.

The asymmetry framework offers one way to interpret the apparently conflicting findings. Early dysfunction after *de novo* DSA in Brooks et al. and late antibodies without uniform graft loss in Pouliquen et al. can coexist if pathogenicity varies with timing, complement fixation, antigen specificity, persistence, and concomitant autoimmunity (19, 20). The preformed-DSA cohorts of Piemonti et al. and Maanaoui et al. support individualized evaluation, but they are too small to exclude clinically important risk (**Figure 4**) (18, 22). This framework is conceptual rather than prospectively validated.

Clinically, this distinction supports active longitudinal surveillance rather than a binary pretransplant exclusion rule. Low-level, non-complement-binding preformed DSA to an otherwise excellent donor may be acceptable after multidisciplinary review. Persistent class II *de novo* DSA, rising PRA, declining C-peptide level, worsening HbA1c, recurrent hypoglycemia, or inflammatory graft dysfunction should prompt reassessment of immunosuppression exposure, adherence, infection, autoimmunity, and repeat-transplant strategy (**Table 4**).

**Table 4:** Conceptual comparison of preformed and *de novo* DSA in pancreatic islet transplantation.

Characteristic	Preformed DSA	*De novo* DSA
*Mechanism*	Recipient memory antibody present before infusion; potential immediate binding to donor HLA if target is accessible	New adaptive response after antigen exposure, inflammation, repeat infusion, or immunosuppression reduction
*Timing*	Detected before transplant or in historic sera	Detected after transplant; may be early with graft dysfunction or late during chronic attrition
*Class predominance*	Depends on sensitizing event; class I and II possible	Class II/DQ may be particularly important, by analogy with kidney and lung transplantation
*Complement activation*	Clinically concerning if complement-binding and high strength, but islet-specific thresholds unknown	May identify pathogenic subset; rarely standardized in islet cohorts
*Clinical consequences*	Inconsistent association with insulin independence or graft survival in available islet data	More consistent association with graft dysfunction, graft loss, or future sensitization, but heterogeneous
*Evidence strength*	Low to moderate; largest focused cohort did not show strong adverse effect	Moderate for adverse signal; still limited by small cohorts and confounding
*Clinical response*	Individualized donor selection, virtual crossmatch, avoid strong complement-binding DSA when feasible	Longitudinal surveillance; evaluate adherence, immunosuppression, autoimmunity, and future transplant strategy

### 2.6 Lessons from solid-organ transplantation

Kidney transplantation provides the strongest evidence that preformed DSA matter. Pre-existing DSA are associated with higher rates of ABMR and inferior long-term graft survival, and complement-binding DSA identify an especially high-risk phenotype (36, 37). *De novo* DSA likewise correlate with chronic ABMR, transplant glomerulopathy, and progressive graft loss (34, 35, 39). These observations are biologically coherent because the kidney allograft exposes donor endothelium throughout a large microvascular network, making antibody-endothelium interaction central to rejection.

Heart, lung, liver, and pancreas transplantation show related but organ-specific patterns. Heart transplantation links DSA with ABMR, cardiac allograft vasculopathy, and mortality (40). Lung transplantation is particularly sensitive to class II/DQ *de novo* DSA, which are associated with chronic lung allograft dysfunction (38). Liver transplantation has historically been considered more resistant to humoral injury, yet *de novo* DSA are associated with worse outcomes in selected cohorts (42). The implication is not that DSA have uniform meaning across organs, but that organ architecture, endothelial phenotype, complement regulation, antigen burden, and immunosuppression determine the translation of antibody into injury.

**Figure 5:**
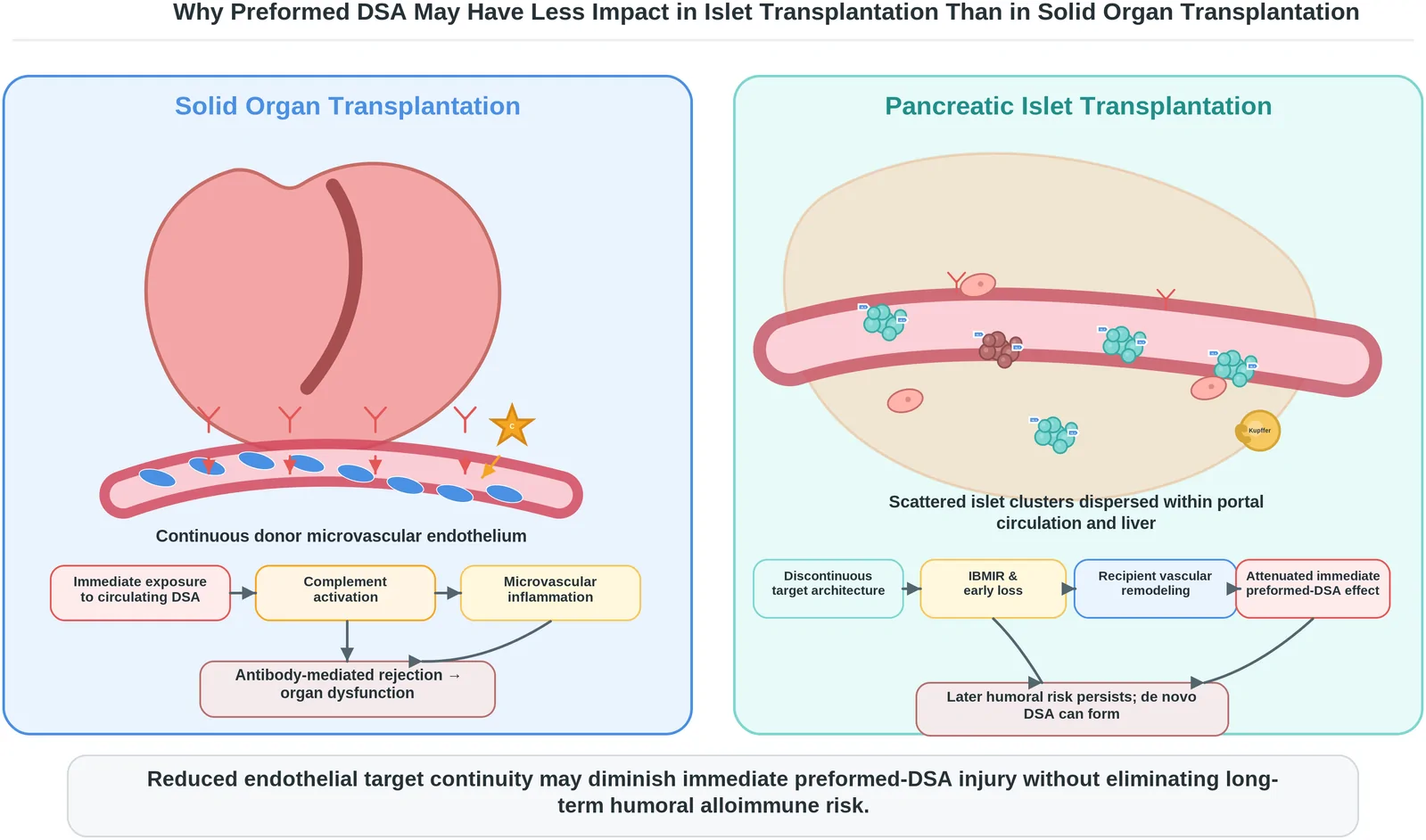
Original schematic comparing vascularized solid-organ and pancreatic islet transplantation. Differences in endothelial target continuity are evidence-supported; the proposed attenuation of immediate preformed-DSA injury in islet grafts is an author-derived conceptual model.

Islet transplantation occupies a distinct mechanistic position because it lacks a continuous donor vascular anatomy, is exposed to portal blood as dispersed cell clusters, undergoes profound early non-adaptive attrition, and may develop recipient-derived vascular support (14–17, 33). These features have been proposed to weaken the immediate effect of some preformed DSA while preserving the importance of adaptive antibody formation over time. This remains a mechanistic model rather than direct proof. Therefore, solid-organ DSA literature should inform, but not dictate, islet allocation (**Figure 5**).

### 2.7 Special situations

Repeat islet transplantation is both therapeutically useful and immunologically complex. Additional infusions can increase islet mass and restore insulin independence, but each transplantation increases HLA exposure and may promote *de novo* alloantibody formation or reactivation of pre-existing alloimmune memory. Donor selection should therefore avoid repeated unacceptable mismatches when feasible, especially in patients with high PRA or DSA burden (21, 26, 27).

Highly sensitized recipients require individualized review. Excluding all sensitized candidates would deny therapy to some patients, many of whom have limited alternatives. However, sensitization affects not only islet success but also future kidney, pancreas, or cellular-therapy options. For candidates likely to need kidney transplantation, the cost of further sensitization may be substantial. Calculated PRA, historic sera, pregnancy, transfusion, transplant history, class II specificity, complement-binding phenotype, and the likelihood of repeat infusion should be considered before proceeding.

Positive crossmatch and high-MFI DSA are not equivalent. A positive physical crossmatch, strong complement-binding DSA, or multiple class II/DQ DSA should raise concern and may justify deferral or alternative donor selection. Low-level SAB reactivity without complement binding and without a corroborating crossmatch may be clinically tolerable in selected cases, particularly when donor islet quality is high and recipient risk from severe hypoglycemia is immediate (22, 35).

Autoantibodies and autoimmunity complicate interpretation. Recurrent beta-cell autoimmunity and alloimmunity can coexist, and declining C-peptide may reflect inadequate engrafted mass, drug toxicity, recurrent autoimmunity, cellular rejection, humoral injury, or non-immune metabolic stress (18, 30, 41). Simultaneous or sequential kidney-islet recipients differ from islet-alone recipients because maintenance immunosuppression may reduce sensitization but kidney graft protection may constrain immunologic risk-taking (27, 43).

Stem-cell-derived islets, encapsulated beta-cell products, and xenotransplantation will reopen the DSA question. Allogeneic stem-cell-derived islets may express HLA and become targets of both pre-existing and *de novo* anti-HLA responses unless immune-evasive engineering or encapsulation succeeds (44–46). Xenogeneic islets introduce non-HLA antibodies, complement activation, and innate immune barriers. The conceptual framework from human allogeneic islets, separating pre-existing antibody from post-engraftment antibody evolution, should be incorporated early into these programs.

### 2.8 Current immunosuppression strategies and humoral-risk modification

Modern islet immunosuppression balances engraftment, beta-cell toxicity, infection, malignancy, kidney function, and alloimmune control. Tacrolimus and sirolimus defined the steroid-free Edmonton era, but calcineurin inhibitors can impair beta-cell function and renal outcomes. Mycophenolate is widely used, while lymphocyte-depleting induction with antithymocyte globulin or alemtuzumab has been used to improve engraftment and reduce early cellular immunity (4, 43, 47). Costimulation blockade and anti-inflammatory biologics have also been explored to improve islet survival and reduce immunologic toxicity (47, 48).

B-cell and plasma-cell targeting are attractive but underdeveloped in islet transplantation. Rituximab and intravenous immunoglobulin were used in the humoral rejection case reported by Kessler et al., but there is no available evidence from randomized studies defining treatment thresholds or benefit (41). Proteasome inhibitors, anti-CD38 antibodies, interleukin-6 pathway blockade, complement inhibitors, and desensitization regimens remain extrapolated from solid-organ transplantation and should not be adopted reflexively without islet-specific safety data.

Because immunosuppression withdrawal is a major trigger for sensitization after graft failure, discontinuation should be planned rather than passive. If graft function is failing but future transplantation remains possible, clinicians should weigh infection and malignancy risks against the risk of durable alloantibody formation and loss of donor options (21). Longitudinal DSA monitoring should be paired with metabolic testing, including fasting and stimulated C-peptide, HbA1c, insulin dose, glucose variability, and severe hypoglycemia frequency.

No islet-specific consensus monitoring schedule has been established. In current practice, DSA assessment is generally anchored to baseline and historic sera, repeated after additional donor infusions and during planned follow-up, and repeated when graft function declines, immunosuppression is reduced or withdrawn, or retransplantation is considered. Serial antibody results should be interpreted alongside crossmatch or complement-binding data when available and the metabolic measures listed above.

## 3 Discussion

Only a limited number of studies directly evaluate preformed DSA, and none is sufficiently large or standardized to define a safe threshold. The absence of a consistent adverse association, therefore, should not be interpreted as equivalence or safety. Although the *de novo* DSA literature is more extensive, it remains observational and cannot establish causality. Evidence specific to preformed DSA is even more limited; therefore, the central clinical inference should be considered provisional rather than definitive.

The current evidence base remains methodologically limited. Most studies are small, single-center, retrospective, or registry-derived. DSA definitions vary, MFI thresholds differ, complement-binding assays are inconsistently used, and many studies span eras of changing immunosuppression, islet isolation, HLA typing, and antibody assay sensitivity. These era effects can create apparent between-study differences that reflect assay sensitivity or treatment practice rather than antibody biology, limiting direct comparison or pooled inference. Recipients frequently receive islet cells from multiple donors, making donor-specific assignment and temporal attribution difficult. Outcomes also vary: insulin independence is a stringent but incomplete endpoint; persistent C-peptide and prevention of severe hypoglycemia may be clinically valuable even without insulin independence (5–8, 48).

Confounding is pervasive and must be explicitly acknowledged. DSA-positive candidates may be selected more carefully, receive better donors, undergo closer monitoring, or be transplanted at centers with advanced immunologic expertise. Conversely, *de novo* DSA may emerge because grafts are already failing and immunosuppression is being reduced, making causality bidirectional. Autoimmunity, non-immune islet cell loss, recurrent inflammation, and drug toxicity can mimic rejection. Publication bias is probable, given that atypical DSA-positive successes and overt humoral rejection episodes are more likely to be reported than neutral or equivocal outcomes.

The review also exposes a measurement problem. SAB MFI is treated as numeric, but it is not standardized enough to function as a universal biological threshold. Complement-binding, IgG subclass, antibody persistence, epitope specificity, eplet mismatch, and longitudinal trajectory may be more meaningful than a single pretransplant MFI. This limitation is not unique to islet transplantation, but the smaller and nonvascularized nature of the graft renders oversimplified threshold-based exclusion particularly hazardous (35).

## 4 Future directions

Prospective, multicenter, and harmonized studies are required to definitively establish the clinical significance of preformed DSA in islet transplantation. Each islet cell infusion should be linked to high-resolution donor and recipient HLA typing, baseline and historic sera, unacceptable antigens, physical or virtual crossmatch, SAB MFI with serum treatment protocols, complement-binding assays, IgG subclass when available, and pre-specified *de novo* DSA definitions. Outcomes should include insulin independence, stimulated C-peptide, HbA1c, insulin dose, continuous-glucose-monitoring metrics, severe hypoglycemia, graft survival, graft loss, repeat transplantation, and cPRA evolution, consistent with established beta-cell replacement outcome definitions (49).

Stem-cell-derived and encapsulated islet trials should embed humoral-immunity and DSA monitoring endpoints from the outset. Studies should report product HLA expression, anti-HLA and non-HLA antibody trajectories, the effects of immune-evasive engineering or encapsulation, and whether inflammatory injury alters antigen exposure. Xenogeneic products will require parallel assessment of complement-mediated and non-HLA antibody responses. The central distinction between pre-existing antibody and post-engraftment antibody evolution therefore applies directly to these emerging platforms (44–46) (**Table 5**).

**Table 5:** Knowledge gaps and future directions.

Issue	Current evidence	Unanswered questions	Future research priorities
*Universal MFI threshold*	No validated islet-specific cutoff; thresholds extrapolated from solid organs	What MFI, persistence, and complement-binding profile predicts graft failure?	Prospective multicenter SAB standardization with central adjudication
*Class II/DQ risk*	Biologically plausible and supported by solid-organ literature	Are DQ dnDSA more pathogenic than class I DSA in islet grafts?	High-resolution HLA typing, eplet mismatch, class-specific longitudinal analysis
*Complement-binding DSA*	Underreported in islet studies	Does C1q/C3d positivity identify a treatable phenotype?	Pre-specified complement-binding assays linked to metabolic endpoints
*Repeat transplantation*	Repeated donors increase antigen exposure	How should donor selection balance islet mass against future sensitization?	Allocation algorithms incorporating unacceptable antigens and eplet load
*Immunosuppression withdrawal*	Known trigger for durable sensitization after failure	When is low-dose continuation justified after partial/failed graft function?	Risk models weighing infection, malignancy, and future transplantability
*Biomarkers beyond DSA*	C-peptide and HbA1c are late and non-specific	Can donor-derived cell-free DNA or immune profiling detect early rejection?	Integrate biomarkers with CGM, MMTT, and DSA kinetics
*Stem-cell-derived islets*	Emerging field with incomplete humoral-risk data	Will HLA-edited or encapsulated products prevent DSA-mediated injury?	Embed DSA and alloimmune endpoints into early-phase trials
*Xenotransplantation*	Non-HLA antibodies and complement likely central	How will anti-pig and anti-carbohydrate antibodies interact with innate immunity?	Parallel humoral monitoring for non-HLA and complement responses

## 5 Conclusion

Evidence directly evaluating preformed donor-specific antibodies (DSA) in pancreatic islet transplantation remains limited and does not establish a universally safe antibody threshold. Current data neither justify automatic exclusion based on preformed DSA alone nor establish that such antibodies are safe. Accordingly, no definitive causal or threshold-based recommendation can be made. Strong, persistent, complement-binding, crossmatch-positive, or class II/DQ DSA should prompt caution, donor reassessment, and individualized multidisciplinary review.

In contrast, *de novo* DSA appear to represent a distinct post-transplant risk signal, as they may reflect active alloimmune recognition and coincide with graft dysfunction, although not all *de novo* DSA are immediately pathogenic. A pragmatic approach should therefore combine individualized pretransplant risk stratification with longitudinal antibody and metabolic surveillance. When evaluating early islet graft dysfunction, humoral risk should be considered alongside other relevant mechanisms, including the instant blood-mediated inflammatory reaction (IBMIR), the hepatic microenvironment, limited transplanted tissue mass, endothelial remodeling, repeat donor exposure, and post-failure sensitization.

## Supplementary material

Supplementary Figure 1 Study identification and selection for the focused narrative review. A focused PubMed search combining islet transplantation with anti-HLA antibody, donor-specific antibody, and sensitization terms (January 2000-June 2026) identified 104 records. Title and abstract screening excluded 76 records (non-human or experimental-model studies, reviews or editorials without primary data, and records without anti-HLA antibody, DSA, PRA, or sensitization outcomes in islet recipients); 28 full-text reports were then assessed, of which 17 were excluded (autologous islet transplantation, overlapping or duplicate cohorts, or data reported only in a solid-organ or mechanistic context), leaving 11 direct islet-transplant studies for the evidence synthesis. A further 38 references were cited for mechanistic, solid-organ comparative, and background context (49 references in total). Because this was a focused narrative review, the counts are reproducible from the described search strategy rather than derived from a prospectively maintained screening log.
